# Comparative Evaluation of Inductively Coupled Plasma Mass Spectrometry (ICP-MS) and X-Ray Fluorescence (XRF) Analysis Techniques for Screening Potentially Toxic Elements in Soil

**DOI:** 10.3390/toxics13040314

**Published:** 2025-04-18

**Authors:** Ilaria Guagliardi, Nicola Ricca, Domenico Cicchella

**Affiliations:** 1 Institute for Agriculture and Forest Systems in the Mediterranean (CNR-ISAFOM), National Research Council of Italy, 87036 Rende, Italy; nicola.ricca@cnr.it; 2 Department of Science and Technology, University of Sannio, 82100 Benevento, Italy; domenico.cicchella@unisannio.it

**Keywords:** inductively coupled plasma mass spectrometry (ICP-MS), X-ray fluorescence (XRF), soil, analytical difference

## Abstract

Soil contamination by potentially toxic elements (PTEs) poses a major environmental concern. The distribution and concentration of these elements can vary significantly in polluted areas, making detailed assessments crucial. A comprehensive analysis is essential to accurately characterise contamination patterns, as a foundation for effective site evaluation and remediation efforts. This study evaluates the effectiveness and reliability of X-ray fluorescence (XRF) and inductively coupled plasma mass spectrometry (ICP-MS) for determining PTEs in soil samples. Statistical analyses reveal significant differences between the two techniques for Sr, Ni, Cr, V, As, and Zn, likely due to variations in detection sensitivity, calibration methods, or matrix effects. Pb exhibits a weaker difference, suggesting a potential, yet statistically insignificant, difference between methods. Correlation analyses indicate a strong linear relationship for Ni and Cr, while Zn and Sr display high variability, limiting direct comparability. Bland–Altman plots highlight systematic biases, particularly for V, where XRF consistently underestimates concentrations compared to ICP-MS. These findings underscore the importance of selecting the appropriate analytical technique based on detection limits, sample characteristics, and measurement reliability. While both methods provide valuable insights for environmental monitoring, carefully considering their limitations is crucial for accurate contamination assessment.

## 1. Introduction

Soil contamination by potentially toxic elements (PTEs) is a significant environmental issue [1]. Polluted areas often show considerable variability in the distribution and concentration of PTEs [2,3]. Therefore, a thorough and detailed area assessment is essential for a comprehensive geospatial understanding of soil contamination [4]. This understanding is a prerequisite for effective site characterisation and reclamation [5,6].

Measuring trace metals is crucial [7], especially in complex environmental matrices that require analytical techniques to provide information about spatially distributed elements and allow examination of their geochemical dynamics [8].

The comparison of screening techniques for PTE assessment is a critical area of research, particularly in environmental monitoring and health risk assessments. Different methods have distinct advantages and limitations that influence their applicability in various contexts [9]. These differences can affect operational principles, sensitivity, and specific application scenarios. Inductively coupled plasma mass spectrometry (ICP-MS) and X-ray fluorescence (XRF) are two of the most commonly used techniques in environmental monitoring, food safety, and health assessments. However, they each have distinct advantages and limitations that determine their suitability for different analytical scenarios.

Inductively coupled plasma mass spectrometry (ICP-MS) is a highly sensitive and precise analytical technique used to quantitatively determine trace and ultra-trace elements in various sample types, including soils, water, and biological materials, providing essential data for risk assessments [10]. It is widely recognised for its exceptional sensitivity and low detection limits, often achieving levels as low as parts per trillion (ppt) for many elements, multi-element analysis, and wide dynamic range. This makes it the preferred method for trace elemental analysis, particularly for PTEs [11,12,13]. It is widely applied in environmental, geological, and industrial analyses due to its ability to detect multiple elements simultaneously with low detection limits. This technique employs high-temperature plasma to ionise the elements, followed by mass spectrometric analysis to quantify the ions produced. The ability of ICP-MS to analyse multiple elements simultaneously [14] and its high throughput capacity are additional advantages that enhance its utility in complex sample matrices [11,15]. For instance, studies have demonstrated that ICP-MS can accurately detect a wide range of elements, including PTEs such as lead, cadmium, and arsenic, with high accuracy and precision [16]. Some limitations can be achieved for matrix effects, sample preparation, which can be time-consuming, instrument cost, and maintenance.

X-ray fluorescence (XRF) is a non-destructive analytical technique that uses X-ray excitation to induce fluorescence in a sample. This process allows identifying and quantifying solid samples’ elemental composition, including soil, rocks, and sediments [17,18], based on their characteristic X-ray emissions. The method relies on the interaction between high-energy X-rays and the sample material to excite atomic electrons and generate characteristic secondary (fluorescent) X-rays. XRF allows for qualitative and quantitative sample composition analysis [19].

While XRF typically has higher detection limits than ICP-MS, it offers rapid analysis and minimal sample preparation advantages. This makes it particularly well suited for field applications and real-time monitoring [20,21]. Recent advancements in XRF technology, such as the development of portable XRF analysers, have further enhanced its applicability in various settings, including environmental assessments and food safety inspections [20,22]. Despite these advantages, XRF has some limitations. The penetration depth for fluorescence emission in soil, particularly for lead, is restricted to the surface layer due to mass absorption coefficients, soil density, and instrument characteristics [23]. As a result, a homogeneous sample is required, which may need to be achieved through acid dissolution into liquids, grinding, or preparing pressed pellets [24]. Additionally, XRF instruments are relatively expensive and less accessible than other techniques, and their detection limits are higher than those of inductively coupled plasma (ICP) methods.

One of the critical differences between these two techniques lies in their sensitivity to sample matrix effects. ICP-MS requires sample digestion, often involving acid treatments, which can introduce uncertainties if the digestion is incomplete or if certain elements are not fully released from the matrix [25]. In contrast, XRF can analyse solid samples directly without extensive preparation, which can be advantageous when dealing with heterogeneous materials or when preserving the integrity of the sample is paramount [21,22]. However, the non-destructive nature of XRF can lead to challenges in quantification, particularly for elements present at low concentrations, as the technique may suffer from matrix effects that can skew results [21].

Moreover, the operational costs associated with each technique differ significantly. ICP-MS instruments are typically more expensive and require skilled personnel for operation and maintenance, which can limit their accessibility for routine analyses [12,22]. On the other hand, XRF instruments, especially portable versions, are generally more affordable and user-friendly, allowing for broader adoption in various sectors, including environmental monitoring and public health [20,21]. This cost-effectiveness makes XRF an attractive option for preliminary screening, where rapid results are needed, and the highest precision is not critical.

In terms of elemental coverage, both techniques have their strengths. ICP-MS can analyse a wide range of elements, including those difficult to detect with XRF, such as certain lanthanides and actinides [11,12]. Conversely, XRF can measure a broader spectrum of elements in a single analysis, which can be particularly useful in applications where multiple elements must be assessed simultaneously [21,22]. However, the elements analysed can vary based on calibration standards and instrument settings, which necessitates careful method validation for accurate quantification [26].

The integration of both techniques can also provide complementary data, enhancing the overall understanding of PTEs in various matrices. For instance, using XRF for initial screening followed by confirmatory analysis with ICP-MS can help validate results and provide a more comprehensive assessment of elemental concentrations [27]. This multi-method approach is particularly beneficial in complex environmental studies, where the presence of multiple contaminants may complicate the analysis.

The choice between ICP-MS and XRF ultimately depends on specific analytical requirements, including the type of sample, the elements of interest, the required detection limits, and the available resources. For instance, in environmental studies where trace levels of toxic metals are of concern, ICP-MS may be the preferred technique due to its superior sensitivity [11,15]. Conversely, for rapid screening in field conditions or when analysing bulk materials, XRF may be more appropriate due to its speed and ease of use [20,21]. In terms of specific applications, both techniques have been employed to assess the presence of toxic elements in various contexts. For example, studies have utilised ICP-MS to evaluate the concentrations of heavy metals in bone samples, revealing significant disparities in results compared to XRF [28]. Similarly, XRF has been effectively used to screen for toxic elements in consumer products, demonstrating its utility in regulatory contexts [25]. The choice between ICP-MS and XRF often depends on the specific requirements of the analysis, including the desired sensitivity, the nature of the sample, and the available resources.

In conclusion, while ICP-MS and XRF are valuable tools for PTE analysis, their distinct operational characteristics, sensitivity, and application contexts necessitate careful consideration when selecting the appropriate technique for a given analytical task. Integrating both methods in a complementary manner could also be beneficial, allowing for the strengths of each technique to be leveraged in comprehensive analytical workflows.

This study aims to compare the effectiveness and reliability of two analytical techniques—X-ray fluorescence (XRF) and inductively coupled plasma mass spectrometry (ICP-MS)—in determining the concentrations of PTEs in soil samples. Soil from urban and peri-urban areas of the municipalities of Cosenza and Rende, in Calabria, southern Italy, was analysed to assess these methods’ consistency, accuracy, and applicability, providing insights into their suitability for environmental monitoring and contamination assessment.

## 2. Materials and Methods

### 2.1. Study Area

The study area, covering 92 km^2^, is situated in the Cosenza–Rende territory in the Calabria region (southern Italy) and is characterised by high vehicular traffic. It lies within the Crati Basin graben, which the Sila Massif borders to the east and the Coastal Chain to the west [29]. The Crati Basin is an NW-oriented tectonic depression that formed during the Pliocene and has been influenced by faults associated with the horst–graben system [30,31,32].

Geologically, the area features a thick succession of Pliocene sediments, Pleistocene to Holocene alluvial sands, and Miocene carbonate rock outcrops [33,34,35], which overlie a Paleozoic intrusive–metamorphic complex formed by paragneiss, biotite schists, and grey phyllitic schists with quartz, chlorite, and muscovite, which, in some cases, are in the process of weathering. The predominant soil types include Fluvisols, Leptosols, Arenosols, Cambisols, Calcisols, Umbrisols, and Phaeozems [36], as well as Vertisols, Luvisols, Entisols, Inceptisols, Mollisols, and Alfisols [37], as mapped in the Calabria region’s 1:250,000-scale soil map [38].

The area experiences a temperate continental climate, marked by significant daily and annual temperature variations [39]. The local soils primarily derive from sediments originating from the surrounding mountain massifs [40].

### 2.2. Soil Sampling

In this study, a collection of 50 soil samples from both residual and non-residual topsoil across various locations, including gardens, parks, flowerbeds, and agricultural fields within the study area, was performed. Additionally, duplicate pairs from every 10th site, which were then split in the laboratory to create replicates, were collected. Before sampling, surface litter was removed from each site.

Topsoil samples (0–10 cm in depth) were collected from five locations—one at each corner and one in the centre of a 20 × 20 m square—using a hand auger. These samples were combined to create a bulk sample. The collected soil was thoroughly mixed, and any foreign materials, such as roots, stones, pebbles, and gravel, were removed. The final volume of the sample ranged from 1 to 1.5 kg, which was further reduced by half through quartering.

### 2.3. Determination of PTE Contents Using XRF and ICP-MS Techniques

Soil samples were dried at 40 °C in the laboratory to eliminate moisture, ensuring a water-free reference for elemental analysis. The soil was then homogenized and sieved to obtain fine soil particles (≤2 mm). All subsequent analyses were conducted on this fine soil, serving as a standard interstudy comparison reference.

Each prepared soil sample was analysed using X-ray fluorescence spectrometry (XRF) and inductively coupled plasma mass spectrometry (ICP-MS) to determine the concentrations of arsenic (As), chromium (Cr), nickel (Ni), lead (Pb), strontium (Sr), vanadium (V), and zinc (Zn).

In this study, the XRF instrument used was the wave dispersive X-ray fluorescent spectrometer Philips PW 1480 (Amsterdam, The Netherlands), with a rhodium anti-cathode tube. It was calibrated using certified reference materials to ensure accurate quantification, precisely the reference rock powders JA-1 andesite, JB-1a basalt, JF-1 feldspar, and JGb-1 gabbro issued by the Geological Survey of Japan [41]. Fine soil samples were compressed into pellets and placed in the spectrometer, and an X-ray beam was directed at the surface. The emitted fluorescence signals were recorded, and the software processed the spectral data to identify and quantify the elements.

In this study, for the ICP-MS method, PTEs were extracted using microwave-assisted digestion in closed Teflon (TFM) vessels with automated pressure and temperature control. The digestion process employed 14 mL of a ternary acid mixture consisting of 6 mL of perchloric acid (HClO_4_), 5 mL of hydrofluoric acid (HF), and 3 mL of nitric acid (HNO_3_) added to a sample quantity of 0.100 g. After the application of the thermal program, the vessels were cooled and opened, and the digested solutions were transferred into 50 mL PFTE containers and placed on a plate at 200 °C to allow their reduction to gel. An evaporation process was then planned. The dissolution procedure was repeated a second time by adding 2 mL of HClO_4_. After the evaporation of this second phase, 5 mL of HNO_3_ was added, and the containers were then left to cool. The residue is dissolved in 2 mL HNO_3_ and 3–5 mL ultra-pure water, heated at 60–80 °C overnight, transferred to a polycarbonate container, and diluted to 100 mL (1000× dilution of sample) before analysis. Analysis of the soil samples was carried out using an ICP-MS PerkinElmer SCIEX—ELAN 6100 DRC-e (MDS Inc., Concord, ON, Canada). The measurement conditions for ICP-MS are presented in Table 1.

Elemental concentrations were then determined according to established international standard methodologies (Table 2). Accuracy of the data was determined as <3% through analysis of the U.S. Geological Survey standards AGV-1, BCR-1, BR, DR-N, GA, GSP-1, and NIM-G, described in the Geochemical Database for Reference Materials and Isotopic Standards (GeoReM, http://georem.mpch-mainz.gwdg.de/, accessed on 18 December 2024) at the rate of 1 per each batch of 20 samples. Duplicate analyses were included in each batch at a frequency of one in twenty samples. Measurement errors were assessed using the relative standard deviation (<5%), calculated from three random sample replicates.

Single-element standard solutions of Cr, Ni, Pb, V, and Zn with an initial concentration of 10 μg/mL were mixed and used for calibration after appropriate dilution to obtain the following concentrations: 0.5, 1.0, 5.0, 10.0, 25.0, and 50.0 ng/mL.

All solutions were prepared with double deionized water (Millipore Purification System Synergy, Molsheim, France).

### 2.4. Statistical Analyses

Statistical analyses were performed on each dataset. The paired *t*-test was used to compare the means of two related samples to assess whether they had a significant difference. The Wilcoxon signed-rank test was used to determine statistical similarities or differences for non-parametric datasets containing two related samples. Regression analysis was conducted using Microsoft Excel to determine if a relationship existed between datasets.

## 3. Results and Discussion

Table 3 summarises the statistics of the soil samples determined by both the XRF and ICP-MS techniques.

The statistical analysis of elemental distributions reveals that most elements exhibit a right-skewed pattern. This means that while most measurements are clustered around lower values, a few samples show notably higher concentrations. These high-concentration outliers can influence overall variability and must be carefully considered when comparing analytical techniques.

When comparing the two methods, ICP-MS generally shows broader distributions, suggesting more significant measurement variability than XRF [42,43,44,45]. This could be attributed to its higher sensitivity, lower detection limits, or potential matrix effects affecting certain elements. In contrast, XRF measurements tend to be more tightly clustered, exhibiting lower variability but potentially underrepresenting extreme values [43,46]. Notably, XRF demonstrates higher kurtosis for some elements, indicating the presence of more extreme values, while ICP-MS typically has lower variability—except for Zn, which displays significant dispersion, suggesting inconsistencies in its measurement across samples [42].

### 3.1. Specific-Element Variability and Trends

Examining variability in more detail, the coefficient of variation (CV) highlights Pb and Zn as the elements with the highest variability, indicating substantial fluctuations in concentration measurements across both methods. Additionally, Cr stands out with the highest skewness and kurtosis, emphasizing its strongly asymmetric distribution and the presence of extreme values. An observation of individual elements reveals method-specific trends:

- Strontium (Sr): ICP-MS and XRF produce similar distributions, though XRF reports a slightly higher median value.
- Nickel (Ni): The ICP-MS distribution appears more skewed, with a few samples displaying significantly higher concentrations than the rest.
- Chromium (Cr): The XRF distribution has a wider tail on the higher concentration side, suggesting a tendency for overestimation in some samples.
- Vanadium (V): ICP-MS and XRF exhibit noticeable differences, with XRF yielding a tighter distribution and less variability.
- Arsenic (As): XRF measurements appear more uniformly distributed, whereas ICP-MS results include a few higher outliers, suggesting greater sensitivity in detecting lower-concentration variations.
- Lead (Pb): Both methods demonstrate high variability, but ICP-MS reports more extreme values, potentially due to its greater sensitivity at lower concentrations.

The observed higher variability in ICP-MS data may result from its greater sensitivity and lower detection limits, which can amplify measurement fluctuations, particularly for trace elements. Additionally, systematic biases in elements such as Cr and Pb suggest potential influences from factors like calibration differences, instrument-specific detection efficiencies, or matrix effects that alter how elements interact with the measurement process. In contrast, the generally tighter distributions seen in the XRF data imply more reliable measurements, though this reliability could come at the cost of missing some extreme values that ICP-MS captures. This difference in measurement behaviour underscores the importance of understanding each technique’s strengths and limitations when interpreting the results. These findings align with those of previous studies. Significant differences have been reported between ICP-MS and XRF for elements such as strontium, nickel, chromium, arsenic, and zinc, with lead showing a weaker yet notable variation [47,48,49].

### 3.2. Statistical Validation: Paired t-Test and Wilcoxon Signed-Rank Test Analysis

A paired *t*-test was conducted to formally assess whether ICP-MS and XRF produce significantly different measurements. This test evaluates whether the mean differences between paired ICP-MS and XRF values are statistically significant. The calculations were performed between the mean values from each dataset, the standard deviation of each group, and the number of data values. The results of the paired *t*-test are summarized in Table 4. The statistical findings further reinforce the observed trends, confirming that some elements exhibit strong measurement discrepancies between techniques.

The *p*-value is a key statistical metric used to determine whether the differences observed between the XRF and ICP-MS measurements are statistically significant. A *p*-value below 0.05 indicates strong evidence that the differences between the two methods are unlikely to be due to random variation alone, suggesting a statistically significant discrepancy. Conversely, a *p*-value above 0.05 implies that any observed differences could be due to chance, meaning that the two methods yield comparable results for that element.

In this analysis, the elements Sr, Ni, Cr, V, As, and Zn exhibit *p*-values below 0.05, indicating significant differences between the measurements obtained using XRF and ICP-MS. These differences could be attributed to variations in detection sensitivity, calibration methods, or matrix effects specific to each technique [50].

Lead presents a *p*-value of 0.0517, slightly above the conventional significance threshold of 0.05. This suggests that the difference between XRF and ICP-MS measurements for Pb is relatively weak and does not meet the strict criteria for statistical significance. However, given its proximity to the threshold, it may still indicate a potential difference that could be relevant in practical applications, especially if additional factors such as sample heterogeneity or instrumental precision are considered.

Overall, these statistical results highlight method-dependent variations in elemental measurements, emphasizing the importance of understanding the strengths and limitations of each analytical technique when interpreting soil composition data.

The Wilcoxon signed-rank test is a non-parametric statistical method used to determine whether there are significant differences between two related datasets—in this case, ICP-MS and XRF measurements. Unlike parametric tests, such as the paired *t*-test, which assumes normally distributed differences, the Wilcoxon test does not require normality and is, therefore, more reliable when the data exhibit skewness, outliers, or other non-normal characteristics [51,52,53]. This makes it particularly useful for comparing element concentrations when distributions are irregular or highly skewed.

By applying the Wilcoxon signed-rank test and the paired *t*-test, valuable insights can be gained regarding the nature of differences between ICP-MS and XRF measurements. This feature is critical in the context of ICP-MS and XRF measurement comparisons, where underlying data may be influenced by various factors, including measurement noise and variability inherent in analytical procedures [54].

If both *p*-values from the Wilcoxon test and the *t*-test are low (*p* < 0.05), this indicates that ICP-MS and XRF measurements differ systematically, meaning that the observed differences are unlikely to be due to random variation alone. In this case, the two methods may not be interchangeable for certain elements, and the source of systematic discrepancies—such as calibration issues, matrix effects, or differences in sensitivity—should be further investigated.

If the Wilcoxon test is significant (*p* < 0.05), but the paired *t*-test is not, this suggests that the difference between the two methods is likely due to non-normal behaviour in the data rather than a simple shift in mean values. Such a result implies that the dataset contains skewed distributions, outliers, or heavy tails, which influence the Wilcoxon test but not the *t*-test. This outcome underscores the importance of considering distribution shape when comparing analytical techniques.

If neither test is significant (*p* > 0.05), this suggests that ICP-MS and XRF are in strong agreement, with no statistically meaningful differences in their distributions. In such cases, the methods can be considered interchangeable for the elements under study, at least within the given measurement conditions.

### 3.3. Bar Chart

**Figure 1:**
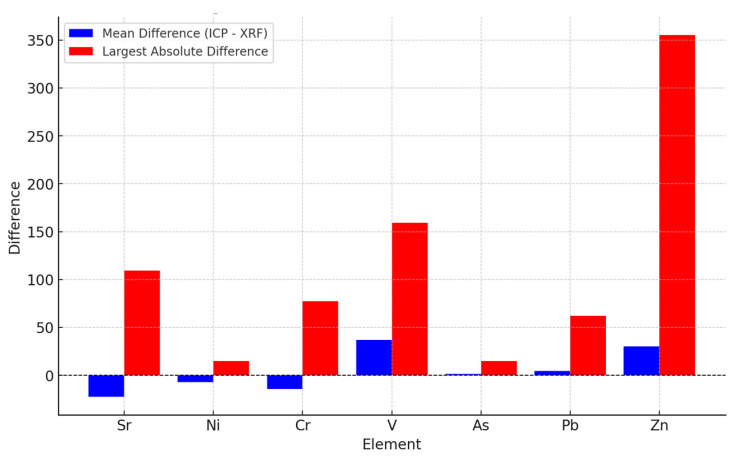
Bar chart values determined using XRF and ICP-MS techniques.

The bar chart comparing the mean differences (ICP-MS—XRF) and the largest absolute difference for each element provides a visual representation of how the two measurement techniques (ICP-MS and XRF) compare in terms of both average differences and extreme values (Figure 1). The chart includes blue bars representing the mean differences and red bars representing the largest absolute differences.

The blue bars reflect the mean differences between each element’s ICP-MS and XRF measurements. These values are calculated by subtracting the XRF measurement from the ICP-MS measurement for each paired observation. Positive mean differences indicate that ICP-MS tends to report higher concentrations than XRF for the respective elements. This could suggest that ICP-MS is more sensitive or has a lower detection limit for that element, leading to higher readings. Negative mean differences suggest that XRF tends to overestimate the concentrations, reporting higher values compared to ICP-MS.

The red bars represent the largest absolute difference observed between the ICP-MS and XRF measurements for each element. This value highlights the maximum difference observed in any individual pair of measurements, providing insight into outliers or extreme variations. Large values for the red bars indicate that while the average difference between the two techniques might be small, there are individual measurements where the difference is much more substantial. These extreme differences may be due to sample heterogeneity, instrumental calibration issues, or matrix effects.

The element-specific observations suggest that the mean difference for V is +36.75, indicating that ICP-MS consistently reports higher concentrations than XRF. This significant positive value suggests that ICP-MS might have better sensitivity for V or is better equipped to detect lower concentrations, leading to its higher values compared to XRF.

Zinc shows the largest absolute difference at 355.22, meaning that there are instances where the differences between ICP-MS and XRF are very large for certain samples. This suggests that some individual samples have extreme variation, potentially due to outliers, instrumental inconsistencies, or matrix effects that impact the measurements from both techniques differently.

Strontium, nickel, and chromium show negative mean differences, indicating that XRF tends to overestimate concentrations compared to ICP-MS. For these elements, the negative values suggest systematic overreporting by XRF, which could be due to factors such as calibration bias, matrix effects, or differences in the sensitivity of the two techniques.

Arsenic and lead show relatively small mean differences, meaning that the overall concentrations measured using ICP-MS and XRF are fairly similar on average. However, there is still some variation, reflected in the differences between individual samples. This suggests that while the methods agree in general, there are still occasional differences that could be due to instrumental or environmental factors influencing the results.

The overall agreement vs. variability suggests that both the general trend (mean difference) and the potential for outliers (absolute difference) make it clear that while ICP-MS and XRF may produce similar results for some elements, there are cases where significant differences arise. Understanding the sources of these differences—whether due to instrument-specific factors, sample heterogeneity, or calibration differences—is essential for making informed decisions about which method to use for each element [55,56].

### 3.4. Regression Trends

**Figure 2:**
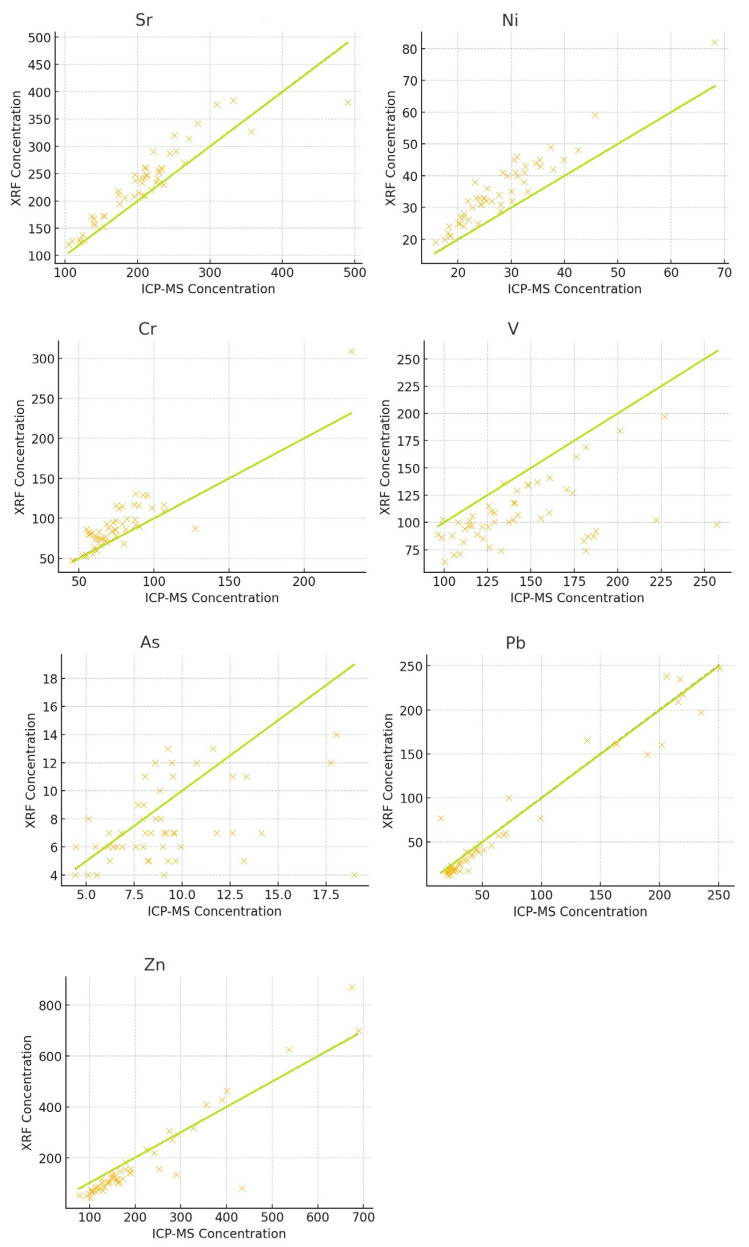
Scatter plots.

The scatter plots in Figure 2 visually represent the relationship between the measurements obtained using ICP-MS and XRF for each element, performed by a Pearson correlation analysis, whose results are reported in Table 5. Each point on the plot corresponds to a paired observation for a specific element, with the x-axis representing the XRF measurement and the y-axis representing the ICP-MS measurement. The green line in each plot represents the ideal 1:1 correlation, where the measurements from both techniques would be identical, meaning that for any given value of XRF, ICP-MS would report the same value. The closer the data points are to this line, the stronger the agreement between the two techniques.

Some elements, such as Ni and Cr, show a clear linear relationship between ICP-MS and XRF measurements, meaning that the two methods tend to produce similar results across the measured concentration range. In these cases, the data points closely follow the 1:1 line, suggesting that there is a good correlation between the two techniques.

For elements like Zn and Sr, the scatter plots reveal significant dispersion (scatter of points), indicating high variability between the measurements obtained using ICP-MS and XRF. This suggests that while the two methods might provide some consistent readings, there are substantial fluctuations and discrepancies in the measurements for these elements, reducing the reliability of direct comparisons.

In the case of V, there is an observable trend where XRF underestimates concentrations compared to ICP-MS. The data points for V tend to fall below the 1:1 line, suggesting that XRF consistently reports lower concentrations than ICP-MS for this element.

### 3.5. Correlation Analysis

Table 4 shows the Pearson correlation analysis results. The correlation coefficient (r), which ranges from −1 to +1, quantifies the degree of association between the two methods: r = 1 indicates perfect positive correlation, r = −1 indicates perfect negative correlation, and r = 0 indicates no correlation.

For Ni, Pb, Zn, Sr, and Cr, the correlation coefficients exceed 0.90, suggesting a very strong correlation between ICP-MS and XRF and highlighting that both ICP-MS and XRF perform similarly in terms of precision and accuracy. This strong relationship indicates that one method does not systematically overestimate or underestimate the concentrations compared to the other. Given the strong correlation for these elements, it may be possible to use either ICP-MS or XRF interchangeably when measuring them, without a significant risk of differences. The strong relationship also suggests that the methods likely measure the same underlying phenomenon, with any differences likely being within acceptable limits for analytical purposes.

For V and As, the correlation coefficients are below 0.50, indicating a weaker correlation between ICP-MS and XRF, implying that the measurements from ICP-MS and XRF do not consistently align. This inconsistency may suggest that either or both techniques are influenced by factors that are not equally addressed by the two methods. These differences could arise from differences in sensitivity, detection limits, or matrix effects specific to these elements. The weak correlation between ICP-MS and XRF for V and As may indicate that one method tends to underestimate or overestimate the concentration of these elements compared to the other. In this case, the choice of method may need to be based on the specific requirements of the analysis. For example, if high precision is critical, it may be important to rely on the method that shows the most consistent results for these elements, or further optimization and validation might be required to align the two methods.

### 3.6. Bland–Altman Plot Analysis

The Bland–Altman plot is a powerful tool for assessing the agreement between the two measurement techniques. Rather than simply evaluating correlation, it provides a visual representation of systematic biases and identifies outliers where the two methods diverge significantly [57].

In the Bland–Altman plot, the x-axis represents the mean concentration for each sample, calculated as

$$Mean=\frac{ICP\text{-}MS+XRF}{2}$$

The y-axis represents the difference between ICP-MS and XRF for each sample:

Difference=ICP-MS−XRF

**Figure 3:**
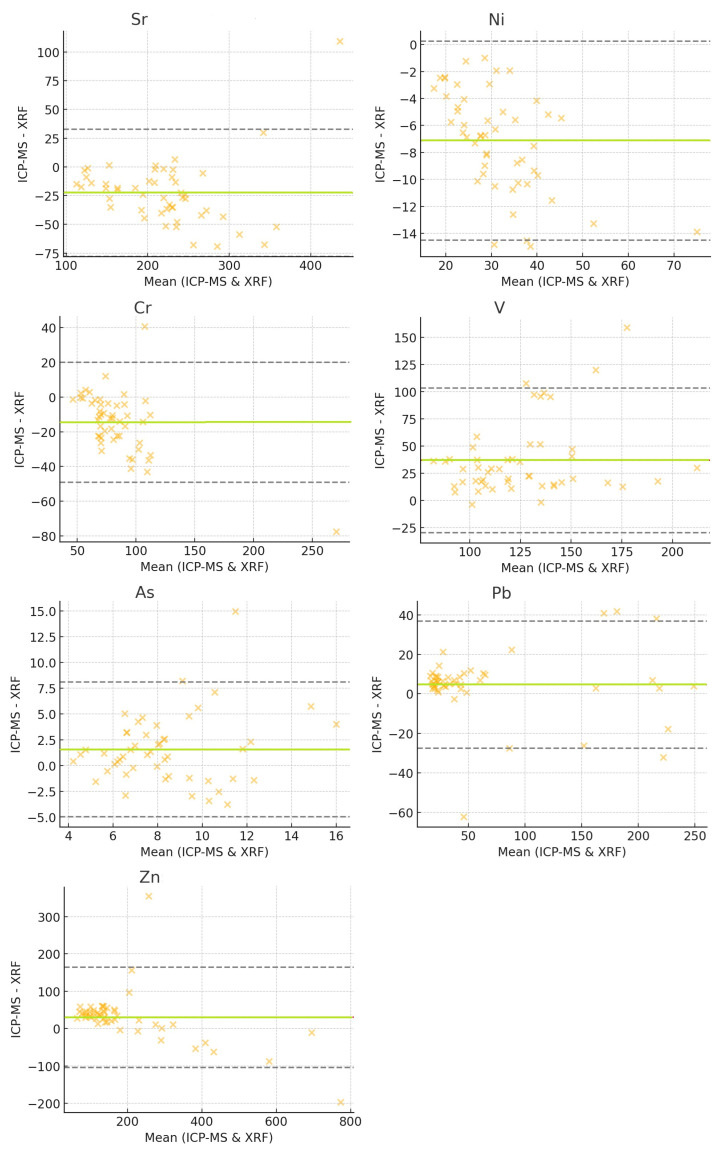
Bland–Altman plots.

In the Bland–Altman plot shown in Figure 3, the green line represents the mean difference, indicating whether one method systematically reports higher values than the other. The grey dashed lines represent the limits of agreement (±1.96 standard deviations from the mean), capturing 95% of the data. Points outside this range are considered outliers, representing significant discrepancies between the two techniques.

Certain samples exhibit differences exceeding ±1.96 standard deviations, suggesting major disagreements between ICP-MS and XRF. These outliers may arise from matrix effects, instrument limitations, calibration errors, or sample inhomogeneity.

For Sr,

- the sample CSRN137 exhibited an extreme difference of +109.39, with ICP-MS reporting 490.39 and XRF reporting 381.

For Ni,

- the samples CSRN020, CSRN024, and CSRN126 showed a ~15 unit lower value in ICP-MS compared to XRF.

For Cr,

- the sample CSRN074 showed an ICP-MS value of 231.56 and an XRF value of 309 (−77.44 difference)
- the sample CSRN097 showed an ICP-MS value of 127.61 vs. an XRF value of 87 (+40.61 difference).

For V, the largest outliers were represented by

- the sample CSRN135, with a +159.03 difference (ICP-MS 257.03 vs. XRF 98),
- the sample CSRN042, with a +107.58 difference,
- the sample CSRN133 with a +120.03 difference.

As displayed, the largest difference was for

- the sample CSRN042 (+14.98), with ICP-MS 18.97 and XRF 4.

Pb,

- with the sample CSRN113 showed a difference of -62.15, with ICP-MS being 14.85 and XRF 77.

Zn exhibited an extreme outlier with

- the sample CSRN067: a difference of +355.22, with being ICP-MS 434.22 and XRF 79.

Several factors could explain the observed differences and outliers between ICP-MS and XRF measurements.

The ICP-MS technique is highly sensitive but subject to matrix suppression, where certain sample components interfere with ionization efficiency. In contrast, the XRF technique is a surface-sensitive technique, meaning that non-homogeneous samples can lead to errors.

Moreover, element-specific interference can occur; for example, for vanadium, ICP-MS consistently reports much higher values than XRF since XRF may not efficiently detect V at low concentrations due to its excitation energy constraints. For nickel, for some samples, XRF consistently reports higher values than ICP-MS. This can be due to background correction errors in XRF affecting Ni detection. XRF may underestimate vanadium concentrations due to its low sensitivity in certain energy ranges.

In addition, XRF analyses only the surface layer, while ICP-MS analyses a fully digested sample. If a sample is not ground evenly, different elemental distributions may be measured using each technique.

XRF has higher detection limits than ICP-MS, making it less sensitive to low concentrations. Background noise in XRF can cause misclassification of elements.

The Bland–Altman approach focuses on individual sample-level agreement, identifying whether measurements from XRF and ICP-MS consistently align. On the other hand, the *t*-test method evaluates differences across the entire dataset rather than at the sample level. Outliers influence Bland–Altman more strongly, as it highlights large deviations in specific cases, while the *t*-test accounts for overall distribution trends, capturing method-dependent variations more broadly. Thus, while Bland–Altman suggests that the methods generally agree, it also reveals cases of major discrepancies. Meanwhile, the *t*-test demonstrates that despite individual agreements, systematic biases exist, reinforcing the need to carefully interpret the results based on the strengths and limitations of each technique.

## 4. Conclusions

This study compared the effectiveness and reliability of X-ray fluorescence (XRF) and inductively coupled plasma mass spectrometry (ICP-MS) for determining potentially toxic elements in soil samples from urban and peri-urban areas of Cosenza and Rende, Calabria, southern Italy. The analysis revealed both the strengths and limitations of each technique, emphasizing their suitability for different applications in environmental monitoring and contamination assessment.

ICP-MS demonstrated higher sensitivity and a broader dynamic range, making it more effective for detecting low concentrations of PTEs. However, it also exhibited greater variability, likely due to matrix effects and instrumental calibration differences. XRF, while providing rapid and non-destructive analysis, tended to underestimate certain elements (e.g., V) and exhibited lower accuracy in highly heterogeneous samples. The statistical analysis showed significant differences between the two methods for Sr, Ni, Cr, V, As, and Zn, while Pb presented a weaker but notable discrepancy.

These findings emphasise the importance of understanding the limitations and strengths of each technique when interpreting soil composition data. While ICP-MS and XRF may provide comparable results for some elements, significant differences arise in others, particularly due to matrix effects, calibration inconsistencies, and instrumental sensitivities. Therefore, careful consideration should be given to the choice of analytical method depending on the element of interest, ensuring accurate and reliable assessments of elemental concentrations in environmental and geochemical studies.

## Figures and Tables

**Table 1:** Measurement conditions for ICP-MS (Perkin-Elmer Sciex ELAN 6100 DRC-e).

ICP-MS Parameter	Value
RF power—W	1200
Argon plasma gas flow—L min^−1^	15
Nebulizer gas flow—L min^−1^	0.82–0.86
Auxiliary gas flow—L min^−1^	1.15
Lens voltage—V	6.00
Nebulizer	Cross flow
Plasma torch	quartz
Sample uptake—mL min^−1^	1
Scanning mode	Peak hop
Dwell time—ms	50
Sweeps/Reading	20
Number of replicates	3
Read delay time—s	15
Cell gas	CH_4_
DRC gas flow—mL min^−1^	0.7
RPq (rejection parameter q)	0.65
Isotope ratio precision (RSD for Ag-107/Ag-109)	<0.2%
LOD—µg kg^−1^	
Sr	0.24
Ni	0.27
Cr	5.02
V	1.9
As	0.7
Pb	0.31
Zn	2.5

**Table 2:** Mean concentrations (mg kg^−1^) obtained for the U.S. Geological Survey standards.

U.S.G.S. Standards	Sr	Ni	Cr	V	As	Pb	Zn
AGV-1	0.51	0.01	0.008	0.12	0.09	0.03	0.08
BCR-1	0.31	0.01	0.13	0.4	0.61	0.01	0.12
BR	1.32	0.2	0.03	0.32	0.02	0.05	0.16
DR-N	0.4	0.01	0.04	0.22	0.003	0.05	0.14
GA	0.31	0.007	0.02	0.03	0.001	0.03	0.08
GSP-1	0.36	0.008	0.01	0.05	0.0001	0.05	0.1
NIM-G	-	0.02	0.01	0.003	0.02	0.04	0.03
Accuracy (%)	1.1	2.4	2	2.8	1.1	2.8	1.4
RPD (%)	2.3	1.3	1.8	1.1	1.1	2.7	1.5

**Table 3:** Descriptive statistics on PTEs (mg kg^−1^) in soil samples, determined using XRF and ICP-MS techniques.

	**Sr**		**Ni**		**Cr**		**V**	
	**XRF**	**ICP-MS**	**XRF**	**ICP-MS**	**XRF**	**ICP-MS**	**XRF**	**ICP-MS**
N. samples	50	50	50	50	50	50	50	50
Min	120.00	105.19	19.00	15.76	47.00	45.71	64.00	96.48
Max	384.00	490.39	82.00	68.11	309.00	231.56	197.00	257.03
Median	233.50	209.70	33.00	25.93	85.50	72.26	100.50	135.73
Mean	231.42	208.94	35.12	27.99	91.36	76.83	107.18	143.93
St. dev.	67.22	68.79	11.07	8.97	37.84	27.63	28.40	36.29
CV (%)	29	32.9	31.5	32	41.4	36	26.5	25.2
Skewness	0.348	1.447	1.582	1.951	3.859	3.693	1.211	1.009
Kurtosis	−0.203	4.172	4.748	6.250	20.03	17.92	1.456	0.687
Difference mean	−22.48 (ICP < XRF)		−7.12 (ICP < XRF)		−14.53 (ICP < XRF)		+36.75 (ICP > XRF)	
Largest absolute difference	109.39		14.95		77.44		159.03	
	**As**		**Pb**		**Zn**			
	**XRF**	**ICP-MS**	**XRF**	**ICP-MS**	**XRF**	**ICP-MS**		
N. samples	50	50	50	50	50	50		
Min	4.00	4.43	12.00	14.85	43.00	78.36		
Max	14.00	18.98	247.00	250.93	871.00	689.48		
Median	7.00	8.73	32.50	36.29	116.50	160.72		
Mean	7.58	9.16	65.38	70.01	181.40	211.39		
St. dev.	2.74	3.25	71.02	70.97	172.81	138.08		
CV (%)	36.2	35.5	108.6	101.4	95.3	65.3		
Skewness	0.735	1.238	1.467	1.459	2.328	1.986		
Kurtosis	−0.532	1.615	0.673	0.530	5.258	3.697		
Difference mean	+1.58 (ICP > XRF)		+4.63 (ICP > XRF)		+29.99 (ICP > XRF)			
Largest absolute difference	14.98		62.15		355.22			

**Table 4:** Paired *t*-test analysis. The results were calculated as mean ± SD for each technique across all samples.

Element	*t*-Statistic	*p*-Value
Sr	5.63	8.80 × 10^−7^
Ni	13.40	5.22 × 10^−18^
Cr	5.84	4.14 × 10^−7^
V	−7.66	6.39 × 10^−10^
As	−3.35	1.56 × 10^−3^
Pb	−1.99	5.17 × 10^−2^
Zn	−3.10	3.23 × 10^−3^

**Table 5:** Correlation analysis.

Element	Pearson Correlation (r)	*p*-Value
Sr	0.91 (strong)	2.01 × 10^−20^
Ni	0.95 (very strong)	3.65 × 10^−26^
Cr	0.90 (strong)	4.01 × 10^−19^
V	0.47 (moderate)	5.46 × 10^−4^
As	0.39 (weak)	4.69 × 10^−3^
Pb	0.97 (very strong)	2.50 × 10^−32^
Zn	0.93 (very strong)	4.34 × 10^−22^

## Data Availability

The original contributions presented in this study are included in the article; further inquiries can be directed to the corresponding authors.
